# A Dynamic Pose-Testing Technique of Landing Gear Combined Stereo Vision and CAD Digital Model

**DOI:** 10.3390/s25216715

**Published:** 2025-11-03

**Authors:** Wendong Zhang, Xianmin Chen, Baoquan Shi, Yao Li

**Affiliations:** 1 National Key Laboratory of Strength and Structural Integrity, Xi’an 710065, China; dongzi.666@163.com (W.Z.); vitochan@163.com (X.C.); 2 AVIC Aircraft Strength Research Institute of China, Xi’an 710065, China; 3 School of Mechano-Electronic Engineering, Xidian University, Xi’an 710071, China

**Keywords:** landing gear, strength test, dynamic pose, visual measurement, CAD digital model

## Abstract

The landing gear is one of the key components of an aircraft, enduring significant forces during takeoff and landing, and is influenced by various uncertain factors related to its structure. Therefore, conducting strength tests on the landing gear structure to study its ultimate load capacity is of great significance for structural design and analysis. This paper proposes a visual measurement method for dynamic pose of landing gear that combines stereo vision and CAD digital model. The method first establishes a measurement reference in CAD digital model and then uses close-range photogrammetry and binocular stereo vision technology to unify the coordinate system of the physical landing gear model with the measurement coordinate system of CAD model. Finally, during the motion of the landing gear, CAD model and the physical model can be synchronized by tracking a small number of key points, thus obtaining the complete motion state of the landing gear during the test. The experimental results demonstrate that the RMSE of the angle error is less than 0.1°, and the RMSE of the trajectory error is under 0.3 mm. This level of accuracy meets the requirements for pose measurement during the landing gear retraction and extension test. Compared to existing methods, this approach offers greater environmental adaptability, effectively reducing the impact of unfavorable factors such as occlusion during testing. It allows for the retrieval of pose information for any point on the landing gear, including its centroid.

## 1. Introduction

The aircraft landing gear, as one of the key components of an aircraft, bears a huge mechanical load during the takeoff and landing processes. There is a complex interaction between the uncertain factors during takeoff and landing and the structural characteristics of the landing gear [1]. Any structural damage may lead to incalculable losses. According to the statistical analysis of previous accidents, accidents caused by the structure of the landing gear account for more than 66% of all aircraft accidents [2]. This data fully reflects the importance of analyzing the static and dynamic mechanical properties of the landing gear structure. Therefore, studying the ultimate load-bearing capacity of the landing gear structure is of great practical significance for its design and analysis.

Visual measurement technology has been widely applied in the ground and airborne tests of aircraft [3,4,5,6,7] due to its remarkable advantages such as non-contact nature, high measurement accuracy, and real-time performance [8,9]. This technology is not only used to verify the effectiveness of the design but also applied in various aspects [10,11] like fault detection and performance evaluation. For instance, in 2002, the Langley Research Center of the National Aeronautics and Space Administration (NASA) in the United States used cameras arranged in a stereo configuration to track and measure the marker points pasted on the aircraft model. By utilizing the changes in the three-dimensional coordinates of these points, the deformation of the aircraft model under dynamic loads was analyzed [12]. This innovative method has laid the foundation for subsequent research. During the period from 2006 to 2014, the AIM (Advanced In-flight Measurement Techniques) project carried out in Europe [13] successfully developed a series of low-cost and high-efficiency three-dimensional optical real-time deformation measurement tools to meet industrial demands.

From the development history of pose visual measurement, pose visual measurement methods are often closely related to the specific measurement features. The point features of images are the most basic image features for realizing pose measurement, which have completed geometric descriptions and perspective projection geometric constraints. Classical point feature extraction methods obtain extracted point features by identifying significant changes in color or grayscale in images [14,15,16]. Pose visual measurement based on point features [17] requires establishing a matching relationship between these point features and the corresponding features in the 3D CAD model, namely the 2D-3D matching relationship. SoftPOSIT [18] is a classic method for directly and synchronously performing 2D-3D matching and pose solving. However, the problems of difficult selection of feature points, large calculation amount, and insufficient robustness in complex environments still have not been solved. The deep learning-based pose visual measurement method based on point features [19,20] overcomes the above shortcomings, greatly improves robustness, realizes point feature extraction under harsh lighting conditions and occlusion, and can greatly reduce the amount of computation. However, the convergence speed during the training process and the versatility of the training model needs further research.

Point features are relatively sensitive to environmental influencing factors such as occlusion and lighting, which affects the robustness and accuracy of pose measurement. Line features are invariant to lighting changes and image noise, and have strong robustness to occlusion. Therefore, pose visual measurement methods based on line features have been widely studied [21,22]. Line features include straight-line features and curve features presented by object contours. For pose visual measurement methods based on straight-line features, the process can first involve the extraction [23,24,25] and matching [21,22] of straight-line features, and then the Perspective-n-Line (PnL) algorithm is used to solve the target pose. For pose measurement methods based on edge features, pose parameters are optimized through continuous projection iteration until the optimization goal is achieved where the projection of the object’s 3D CAD model completely coincides with the target edge [26,27].

Compared with pose measurement methods based on features such as points and lines, region-based methods can utilize more target surface features, offering stronger robustness and higher accuracy. Early region-based pose measurement methods [28] mainly employed probabilistic statistical methods to construct descriptions of local regions. In recent years, deep learning [29,30] has been gradually introduced into region-based pose measurement methods to extract and represent features, further enhancing the performance of region feature-based pose measurement methods. However, since region-based methods require processing all pixels on the target surface, this reduces the processing speed of such methods. Region feature representation methods that improve processing speed and robustness still require further research.

Using only the single features mentioned above for pose measurement results in poor performance and easy failure in scenarios such as occlusion, lighting changes, and high object symmetry. Therefore, pose visual measurement methods based on multi-feature fusion have been studied. Choi et al. [31,32] proposed an iterative pose estimation method that fuses edge and point features. Feature points are used to estimate the initial pose during the global pose estimation process, and then edge features are used to iteratively optimize the pose during the local pose estimation process. Pauwels et al. [33] utilized a binocular vision approach to perform iterative pose estimation by fusing multi-features such as surface point features, color features, and optical flow information of the measured target. With the development of deep learning technology, Hu et al. [34] applied deep learning to object pose measurement and designed a two-stream network structure. The segmentation stream and regression stream in the network target the region features and point features of images, respectively. By combining these two features, more reliable pose information is output, which can effectively handle multiple mutually occluded objects with poor texture. Zhong et al. [35] proposed a polar coordinate-based local region segmentation method. By using a fusion of region distance features and color features to detect pixels in occluded parts, this method demonstrates good robustness in pose measurement for partially occluded targets.

This paper proposes a visual measurement method for the dynamic pose of the landing gear that combines CAD model and actual test. This method is based on close-range photogrammetry and binocular stereo vision technology, and aims to align the coordinate system of the physical model of the landing gear with the coordinate system of the CAD digital model. By tracking a small number of key points, the CAD digital model is driven to move synchronously with the physical model, so as to obtain the complete motion state of the landing gear during the test. This method significantly reduces the impact caused by factors such as occlusion during the test, enabling the real-time and accurate acquisition of the pose information of the landing gear under different working conditions, which is of great significance for the research and optimization of the landing gear structure.

## 2. Methodology

**Figure 1:**
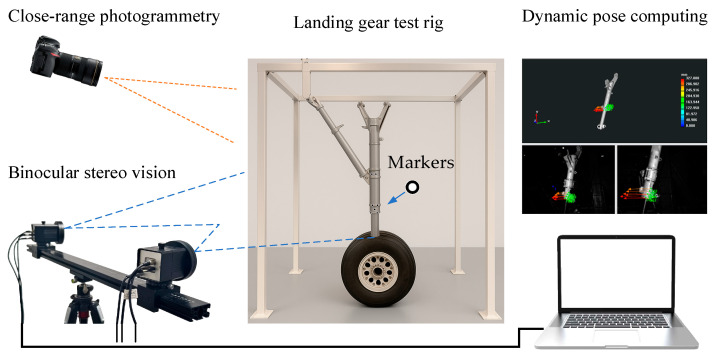
Setup of the Landing Gear Retract and Extend Test.

**Figure 2:**
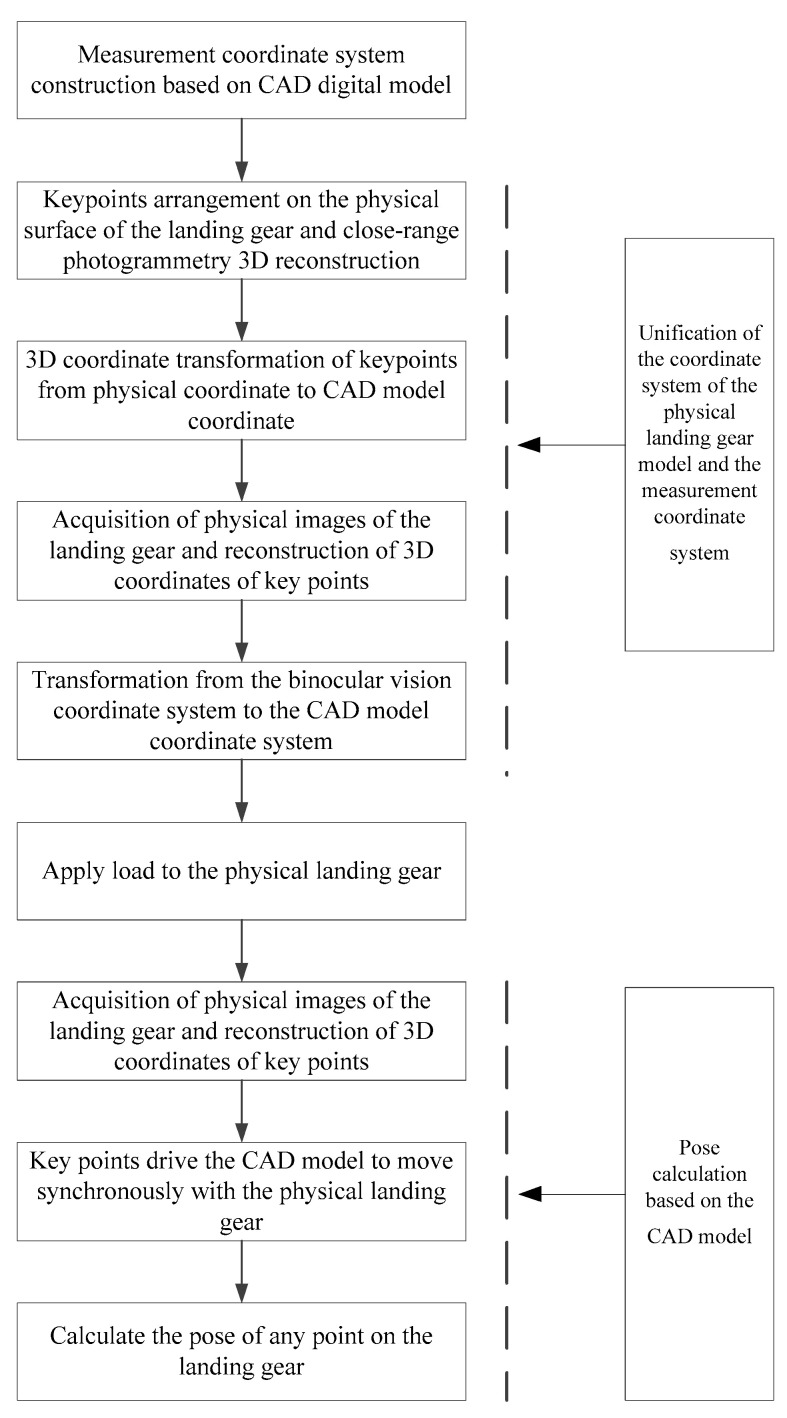
Flowchart of Real-Time Measurement of Landing Gear Pose.

The layout of the landing gear retracts and extend test is shown in Figure 1. The movement space of the landing gear is approximately 2 m × 2 m, and the test frequency is about 0.1 Hz. The measurement process for the dynamic pose of the landing gear, which integrates the CAD digital model with the physical model, is illustrated in Figure 2. Firstly, a measurement coordinate system is established on CAD model, providing a reference benchmark for subsequent coordinate transformation and data processing. Then, by integrating the close-range photogrammetry technology and the binocular stereo vision technology, the coordinate system of the physical model of the landing gear is made consistent with the measurement coordinate system of CAD model. The specific steps include: (1) Arranging a certain number of circular non-coded marker points as key points in the observable area of the physical model of the landing gear; (2) Using photogrammetry technology to reconstruct the 3D coordinates of the key points, and adopting the 3-2-1 coordinate transformation or the feature-based point cloud-CAD model registration method to transform the coordinates of the key points into the measurement coordinate system; (3) Using binocular cameras to collect images of the landing gear and reconstruct the 3D coordinates of the key points; (4) Transforming the binocular vision coordinate system into the coordinate system of CAD model. Finally, after applying a load to the physical model of the landing gear, the binocular stereo vision system tracks and reconstructs the 3D coordinates of the key points in real time (50 Hz), and drives the synchronous movement of CAD digital model through the key points. The software runs on a laptop equipped with an Intel Core i9-14900HX processor, an RTX 5060 Ti GPU, and Windows 11.

### 2.1. Construction of the Measurement Coordinate System Based on CAD Digital Model

The measurement coordinate system serves as the benchmark for determining the pose information. Accurately establishing the measurement coordinate system at the test site has always been a major challenge in visual measurement. This is especially true for irregular measurement objects, as it is difficult to determine an appropriate measurement reference. In this paper, a measurement coordinate system is established on CAD digital model. By arranging key points on the surface of the physical model of the landing gear, the coordinate systems of the digital model and the physical model are registered. In this way, the coordinate system of the physical model is unified with that of CAD digital model, and the consistency of the measurement coordinate system is achieved.

### 2.2. Unification of the Coordinate System of the Physical Landing Gear Model and the Measurement Coordinate System

**Figure 3:**
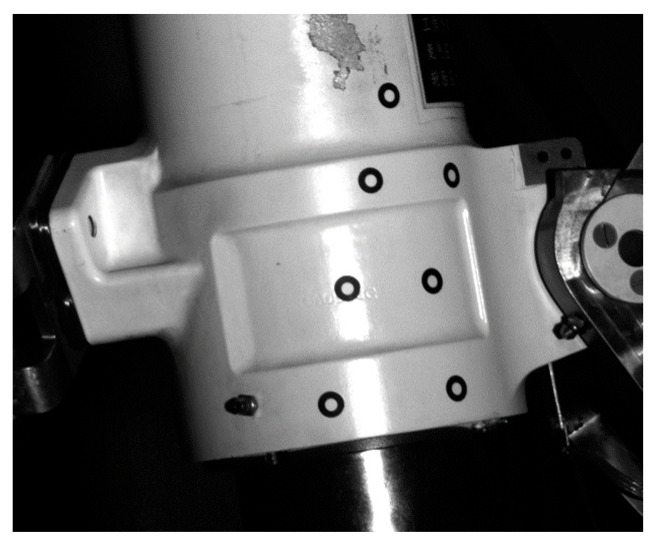
Markers Arranged in Local Areas of the Landing Gear.

The unification of the coordinate system of the physical model of the landing gear and the measurement coordinate system established on CAD digital model is the basis for the correct calculation of the pose. In this paper, the unification of the virtual and real coordinate systems is mainly achieved by arranging several key points (6~10 circular non-coded marker points) on the surface of the physical model of the landing gear. As shown in Figure 3, only a small number (not less than four) of circular non-coded marker points need to be arranged in the local area of the surface of the landing gear to ensure that these marker points can be clearly imaged by the binocular camera throughout the movement process. The marker placement ruler is as follows: (1) The markers are non-collinear; (2) The markers are non-coplanar; (3) All markers must be visible throughout the entire motion of the object. These key points serve as reference points to transform the coordinate system of the physical model of the landing gear into the coordinate system of CAD digital model (i.e., the measurement coordinate system).

#### 2.2.1. Reconstruction of the Three-Dimensional Coordinates of Key Points

To unify the coordinate system of the physical model with the coordinate system of CAD digital model (i.e., the measurement coordinate system), the close-range photogrammetry system is first used to reconstruct the three-dimensional coordinates of the key points arranged on the surface of the physical model of the landing gear, and then these three-dimensional coordinates are transformed into the coordinate system of CAD digital model.

**Figure 4:**
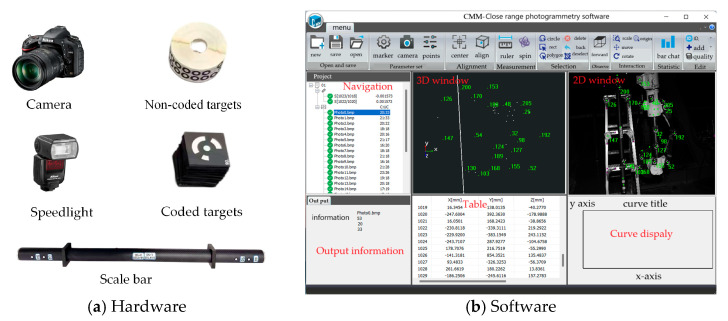
The close-range photogrammetry system.

The hardware and software configurations of the close-range photogrammetry system used are shown in Figure 4. The system hardware includes a Nikon D610 digital single-lens reflex camera, circular coded targets, circular non-coded targets, and a scale bar. The system software, which can run on the Windows platform, includes functions such as calculation mode, deformation mode, and comparison mode.

**Figure 5:**
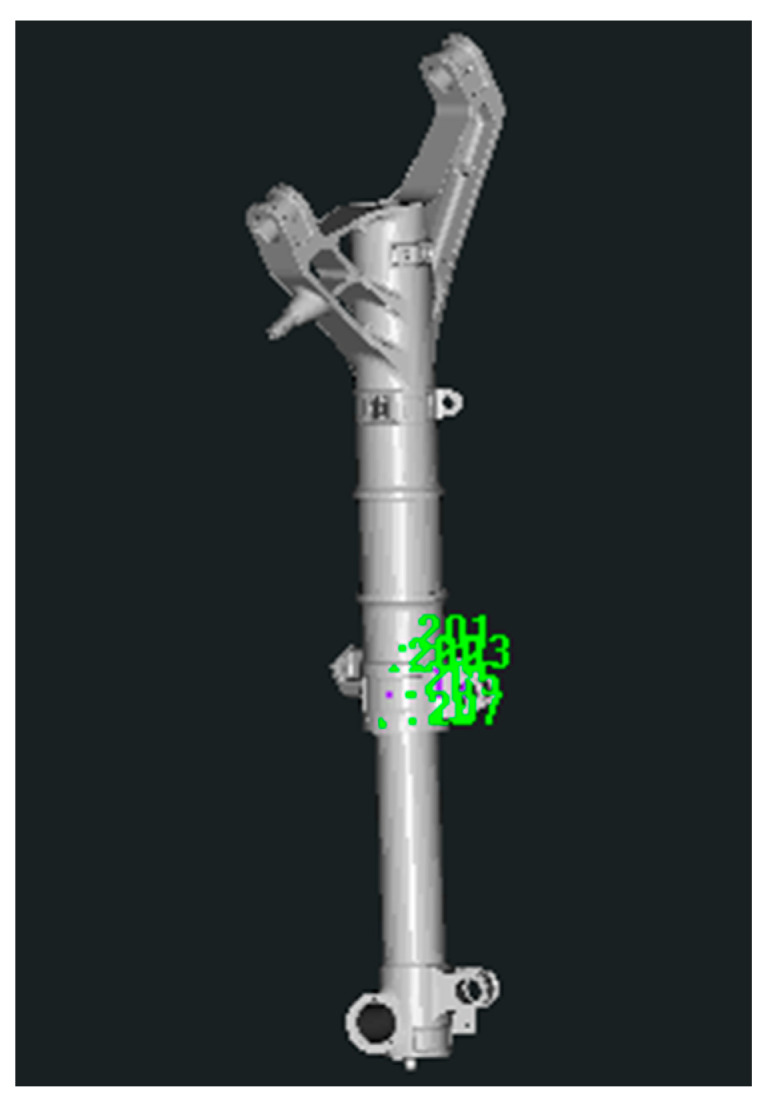
Transformation of Key Points from Close-Range Photogrammetry to the Measurement Coordinate System.

In the process of close-range photogrammetry reconstruction, it is necessary to arrange some coded and non-coded marker points around and on the surface of the landing gear to facilitate relative orientation. In addition, at least one scale bar needs to be placed in the measurement field of view, as shown in Figure 4b. After reconstructing the three-dimensional coordinates of the key points, the 3-2-1 coordinate transformation method or the best fitting method is used to transform the three-dimensional coordinates of these key points into the measurement coordinate system, denoted as $P_{B}^{i}$, 1≤i≤N, *N* represents the number of key points, see Figure 5.

#### 2.2.2. Transformation of the Coordinate System of Binocular Stereo Vision

**Figure 6:**
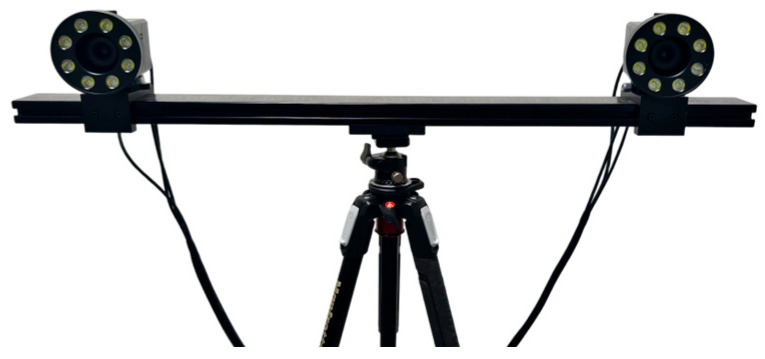
Binocular stereo vision system.

The binocular stereo vision system used is shown in Figure 6. This system consists of two Basler acA2440-75 μm cameras with an image resolution of 2448 × 2048, a pixel size of 3.45 μm, and Computar 16 mm lenses mounted on a crossbeam. Meanwhile, LED light sources are installed on the crossbeam to provide the illumination required for image acquisition. During the measurement process, the binocular cameras are aimed at the landing gear, and the measurement field of view covers the movement range of the marker points on the landing gear.

(1) Calibration of the binocular stereo vision system

Due to the interference of various factors, there will be deviations between the actual positions of image points on the imaging plane and their theoretical positions [36,37]. The factors interfering with imaging mainly include four types of distortions: radial distortion, tangential distortion, image plane distortion, and internal orientation errors of the camera lens. Therefore, camera calibration [38] is required to correct these distortions. In this paper, a cross target is used as the calibration object, and a camera distortion model with ten parameters is adopted to calibrate the binocular cameras.

The radial distortion can be expressed as:

(1) $$\left\{\begin{array}{c} dx_{r}=x(K_{1}r^{2}+K_{2}r^{4}+K_{3}r^{6})\\ dy_{r}=y(K_{1}r^{2}+K_{2}r^{4}+K_{3}r^{6}) \end{array}\right.,$$

The tangential distortion can be expressed as:

(2) $$\left\{\begin{array}{c} dx_{d}=B_{1}(r^{2}+2x^{2})+2B_{2}xy\\ dy_{d}=B_{2}(r^{2}+2y^{2})+2B_{1}xy \end{array}\right.,$$

The image plane distortion can be expressed as:

(3) $$\left\{\begin{array}{l} dx_{m}=E_{1}x+E_{2}y\\ dy_{m}=0 \end{array}\right.,$$

Internal orientation errors of the camera lens can be expressed as:

(4) $$\left\{\begin{array}{c} dx_{n}=\Delta x_{0}+\frac{x}{f}\times \Delta f\\ dy_{n}=\Delta y_{0}+\frac{y}{f}\times \Delta f \end{array}\right..$$

where (x, y) represents the pixel coordinates, $r=\sqrt{x^{2}+y^{2}}$, $K_{1},K_{2},K_{3}$ represents the radial distortion coefficient, $B_{1},B_{2}$ represents the decentering distortion coefficient, $E_{1},E_{2}$ represents the distortion of the image plane, and $\Delta f,x_{0},y_{0}$ represents the error of the internal orientation elements.

Finally, the ten-parameter distortion model can express as

(5) $$\left\{\begin{array}{c} dx=dx_{r}+dx_{d}+dx_{m}+dx_{n}\\ dy=dy_{r}+dy_{d}+dy_{m}+dy_{n} \end{array}\right.,$$

That is,

(6) $$\left\{\begin{array}{l} dx=\frac{x}{f}\Delta f+x_{0}+K_{1}xr^{2}+K_{2}xr^{4}+K_{3}xr^{6}+B_{1}(r^{2}+2x^{2})+2B_{2}xy+E_{1}x+E_{2}y\\ dy=\frac{y}{f}\Delta f+y_{0}+K_{1}yr^{2}+K_{2}yr^{4}+K_{3}yr^{6}+2B_{1}xy+B_{2}(r^{2}+2y^{2}) \end{array}\right.$$

**Figure 7:**
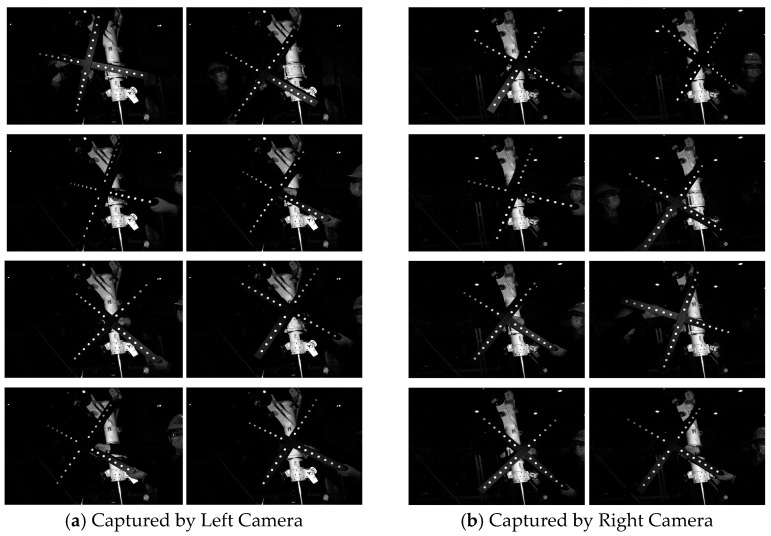
Image of the Cross-Calibration Target Captured by the Stereo Cameras.

To calibrate the system, we captured at least eight cross-target images from different poses using the binocular stereo vision system (Figure 7). The intrinsic and extrinsic parameters of the cameras were then derived from these images via target recognition. The final intrinsic parameters for the left and right cameras are summarized in Table 1.

(2) Coordinate System Transformation of Binocular Stereo Vision

After the calibration is completed, control the binocular cameras to synchronously capture a frame of the physical model image of the landing gear, and reconstruct the three-dimensional coordinates $P_{A}^{i}$ of the N key points arranged on the landing gear. According to the three-dimensional coordinates of the key points in the binocular stereo vision system and their corresponding coordinates in the measurement coordinate system $P_{B}^{i}$ (see Section 2.2.1 for details), calculate the transformation matrix (R,T) to complete the coordinate system transformation.

(7) $$P_{B}^{i}=R\times P_{A}^{i}+T,\;1\leq i\leq N$$

The SVD method can be used to calculate the value of R,T:

(8) $$\left\{\begin{array}{l} H=\sum_{i=1}^{N}\left(P_{A}^{i}-P_{A}^{C}\right){\left(P_{B}^{i}-P_{B}^{C}\right)}^{T}\\ [U,S,V]=SVD(H)\\ R=VU^{T}\\ T=-R\times P_{A}^{C}+P_{B}^{C} \end{array}\right.$$

where ($P_{C}^{A},P_{C}^{B}$) represents the centroid of the point set $P_{A}^{i}$ and $P_{B}^{i}$, $[U,S,V]$ represents the result of singular value decomposition.

### 2.3. Real-Time Calculation of the Dynamic Pose of the Landing Gear

#### 2.3.1. Real-Time Detection and Reconstruction of Key Points

(1) Real-time detection of key points

**Figure 8:**
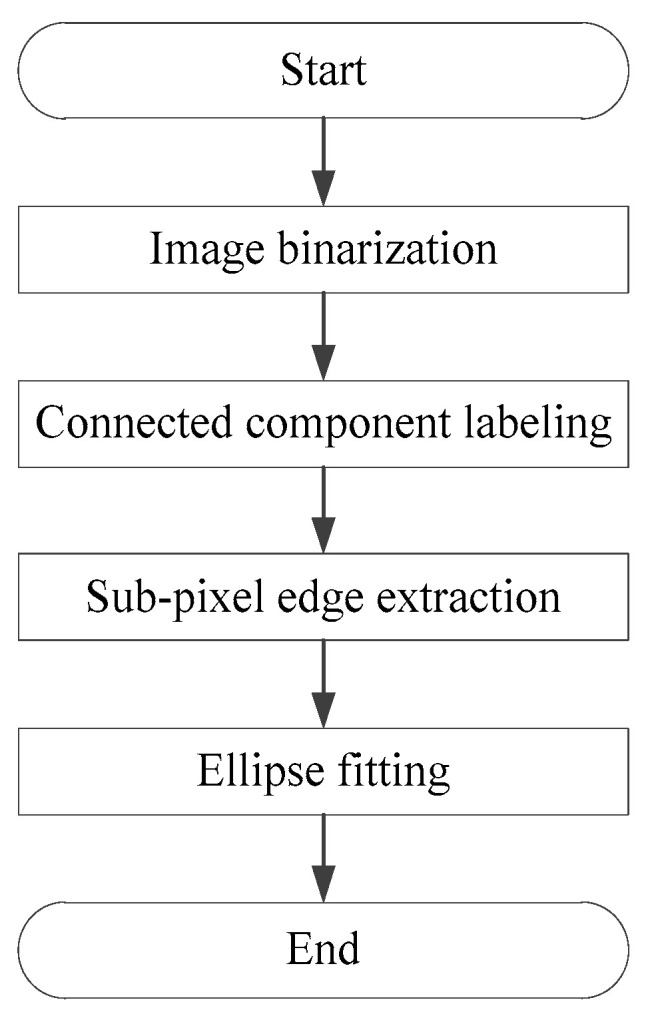
Detection of marker points.

The system detects and identifies marker points placed on the surface of the landing gear, extracting their central coordinates. First, the images captured by the binocular stereo vision system undergo adaptive binary processing to separate the background from the targets to be identified. Next, the connected domains of the targets are marked to obtain the edge information of the marker points. Subsequently, the subpixel extraction of Zernike moments-based edge detection algorithm is performed on the edges of the marker points with high accuracy about 0.1 pixel. Finally, ellipse fitting is used to determine the central coordinates of the marker points. As shown in Figure 8, the overall process of the marker point detection algorithm consists of four steps: image binarization, connected domain marking, subpixel edge extraction, and ellipse fitting. To enhance detection efficiency, GPU programming technology is employed to implement the marker point detection algorithm on the CUDA platform of RTX 5070Ti.

(2) Real-time Reconstruction of 3D Coordinates of Key Points

**Figure 9:**
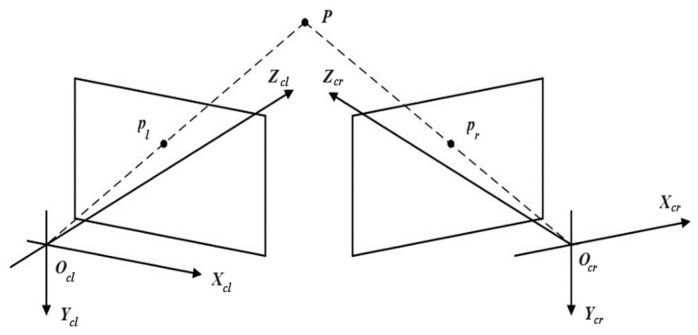
Schematic diagram of the binocular stereo vision model.

The binocular stereo vision model is shown in Figure 9. The coordinates of object point P in the world coordinate system are $\left(x_{w},y_{w},z_{w}\right)$, and the coordinates of its image points $p_{l}$ and $p_{r}$ in the left and right cameras are $\left(u_{l},v_{l}\right)$, $\left(u_{r},v_{r}\right)$ respectively.

Assuming the internal and external parameters of the left and right cameras are ($F_{l}$,${F_{l}}^{\prime }$) and ($F_{r}$,${F_{r}}^{\prime }$) respectively, the following relationship exists:

(9) $$\left\{\begin{array}{l} z_{cl} [\begin{array}{c} u_{l}\\ v_{l}\\ 1 \end{array}]=F_{l}{F_{l}}^{\prime } [\begin{array}{c} x_{w}\\ y_{w}\\ z_{w}\\ 1 \end{array}]= [\begin{array}{cccc} a_{11} & a_{12} & a_{13} & a_{14}\\ a_{21} & a_{22} & a_{23} & a_{24}\\ a_{31} & a_{32} & a_{33} & a_{34} \end{array}] [\begin{array}{c} x_{w}\\ y_{w}\\ z_{w}\\ 1 \end{array}]\\ z_{cr} [\begin{array}{c} u_{r}\\ v_{r}\\ 1 \end{array}]=F_{r}{F_{r}}^{\prime } [\begin{array}{c} x_{w}\\ y_{w}\\ z_{w}\\ 1 \end{array}]= [\begin{array}{cccc} b_{11} & b_{12} & b_{13} & b_{14}\\ b_{21} & b_{22} & b_{23} & b_{24}\\ b_{31} & b_{32} & b_{33} & b_{34} \end{array}] [\begin{array}{c} x_{w}\\ y_{w}\\ z_{w}\\ 1 \end{array}] \end{array}\right.$$

Using the least squares method to solve, the calculation formula for the spatial coordinates of the object point can be derived as:

(10) $$P={\left(A^{T}A\right)}^{-1}A^{T}B$$

Here,

$$\left\{\begin{array}{l} A= [\begin{array}{ccc} a_{11}-a_{31}u_{l} & a_{12}-a_{32}u_{l} & a_{13}-a_{33}u_{l}\\ a_{21}-a_{31}v_{l} & a_{22}-a_{32}v_{l} & a_{23}-a_{33}v_{l}\\ b_{11}-b_{31}u_{r} & b_{12}-b_{32}u_{r} & b_{13}-b_{33}u_{r}\\ b_{21}-b_{31}v_{r} & b_{22}-b_{32}v_{r} & b_{23}-b_{33}v_{r} \end{array}]\\ B= [\begin{array}{c} u_{l}a_{34}-a_{14}\\ v_{l}a_{34}-a_{24}\\ u_{r}b_{34}-b_{14}\\ v_{r}b_{34}-b_{24} \end{array}] \end{array}\right.$$

#### 2.3.2. Dynamic Pose Calculation

Assume that the three-dimensional coordinates of the i-th key point reconstructed from the 0th frame image captured by the binocular camera before and after the landing gear movement are $P_{A}^{0,i}$. During the movement, the coordinates of the i-th key point reconstructed from the j-th frame image captured by the binocular camera are $P_{A}^{j,i}$. Then, the coordinate correspondence of the key points in different frames can be expressed as:

(11) $$P_{A}^{0,i}=R_{j}\times P_{A}^{j,i}+T_{j}$$

The rotation matrix $R_{j}$ and translation matrix $T_{j}$ can be computed using the SVD method described in Section 2.2.2:

(12) $$\left\{\begin{array}{l} R_{j}= [\begin{array}{ccc} r_{j11} & r_{j12} & r_{j13}\\ r_{j21} & r_{j22} & r_{j23}\\ r_{j31} & r_{j32} & r_{j33} \end{array}]\\ T_{j}= [\begin{array}{c} t_{jx}\\ t_{jy}\\ t_{jz} \end{array}] \end{array}\right.$$

By performing Euler angle decomposition on the rotation matrix $R_{j}$, the attitude angles of the landing gear in the j-th frame image can be obtained:

(13) $$\left\{\begin{array}{l} \alpha_{j}=\arctan2\left(\frac{r_{j32}}{r_{j33}}\right)\\ \beta_{j}=-\arcsin\left(r_{j31}\right)\\ \gamma_{j}=\arctan2\left(\frac{r_{j21}}{r_{j11}}\right) \end{array}\right.$$

For the position information of any point $Q_{A}^{j,i}$ on the landing gear in the j-th frame image, excluding the key points, it can be obtained using the following equation:

(14) $$Q_{A}^{j,i}=R_{j}\times Q_{A}^{0,i}+T_{j}$$

Here, $Q_{A}^{0,i}$ represents the initial coordinates of point $Q_{A}^{j,i}$ in CAD model. Since the coordinates of any point on the CAD digital model can be obtained, the pose information of any point on the landing gear can be calculated according to Equation (14).

#### 2.3.3. Covariance Estimation for Pose Data

The problem of propagating uncertainty from 3D key point correspondences to the transformation parameters (rotation ***R*** and translation ***T***)—often estimated via Singular Value Decomposition (SVD)—is frequently addressed in point cloud alignment. This is central to algorithms like Iterative Closest Point (ICP) and feature-based registration [39], which aim to find the relative pose that minimizes the sum of squared distances between reference and sensed points. The covariance of the estimated transformation can be derived as follows:

(15) $$\left\{\begin{array}{l} C=\sum_{i=1}^{n}{\left\|P_{B}^{i}-RP_{A}^{i}-T\right\|}^{2}\\ \mathrm{cov}(l)={\left(\frac{\partial^{2}C}{\partial l^{2}}\right)}^{-1}\left(\frac{\partial^{2}C}{\partial m\partial l}\right)\mathrm{cov}(m){\left(\frac{\partial^{2}C}{\partial m\partial l}\right)}^{T}{\left(\frac{\partial^{2}C}{\partial l^{2}}\right)}^{-1} \end{array}\right.$$

where m represents the n sets of correspondences $\left\{P_{A},P_{B}\right\}$, and ***I*** is a vector of the relative pose expressed as $I= [\alpha ,\beta ,\gamma ,t_{x},t_{y},t_{z}]$, cov(l) is the covariance of the output pose, and cov(m) is the covariance of the input point vector. A detailed derivation is provided in Reference [40]. The Monte Carlo (MC) simulation results [39] demonstrate that when the input correspondence noise (measured as the mean squared error of distances) is below 10^−1^, the resulting covariance of the output pose can reach on the order of 10^−3^ under the given data scale.

## 3. Experimental Results and Discussion

The measurement accuracy of the landing gear’s dynamic pose is primarily determined by the reconstruction accuracy of close-range photogrammetry and the measurement accuracy of binocular stereo vision. Therefore, under laboratory conditions, experimental verifications were respectively conducted on the reconstruction accuracy of key points in close-range photogrammetry, the 3D reconstruction accuracy of binocular stereo vision, and the calculation accuracy of attitude angles.

### 3.1. Evaulation of the Reconstruction Accuracy of Key Points in Close-Range Photogrammetry

**Figure 10:**
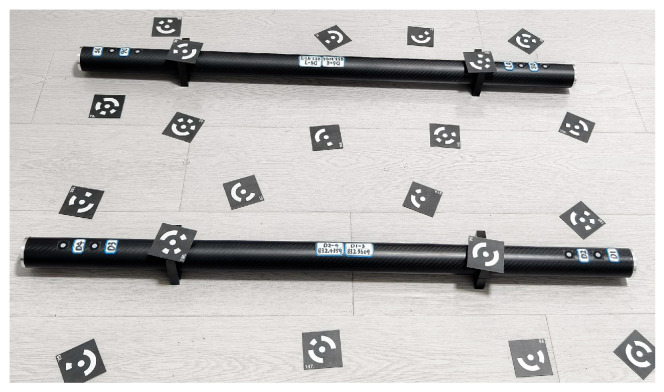
Verification of the accuracy of the close-range photogrammetry system.

In order to verify the reconstruction accuracy of close-range photogrammetry, as shown in Figure 10, two calibrated scale bars are placed in the field of view. One of the scale bars is used as a reference for 3D reconstruction, and then the length of the other scale bar is measured and compared with its calibrated length, so as to obtain the measurement error and the RMSE value. The results are shown in Table 2. The reconstruction accuracy of close-range photogrammetry is better than 0.03 mm.

### 3.2. Evaulation of the Reconstruction Accuracy of Key Points in Binocular Stereo Vision

**Figure 11:**
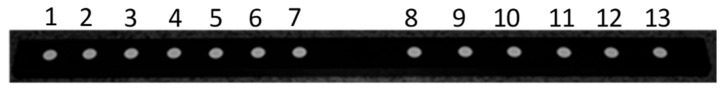
The calibrated bar used for verify the accuracy of the binocular stereo vision system.

The calibrated bar shown in Figure 11 is utilized for verification. This calibrated bar consists of 13 circular non-coded marker points, which serve as key reference points for accuracy assessment. The verification process involves calculating the difference between the distances of marker points reconstructed by the binocular stereo vision system and the corresponding calibrated values. These differences are then statistically analyzed to compute the Root Mean Square Error (RMSE), a standard metric for evaluating the reconstruction accuracy.

The results of this verification process are summarized in Table 3, where it is evident that the reconstruction error of the binocular stereo vision system is less than 0.08 mm. This indicates that the system is capable of achieving highly precise distance measurements, confirming its reliability and accuracy in practical applications.

### 3.3. Evaulation of the Calculation Accuracy of Attitude Angles

**Figure 12:**
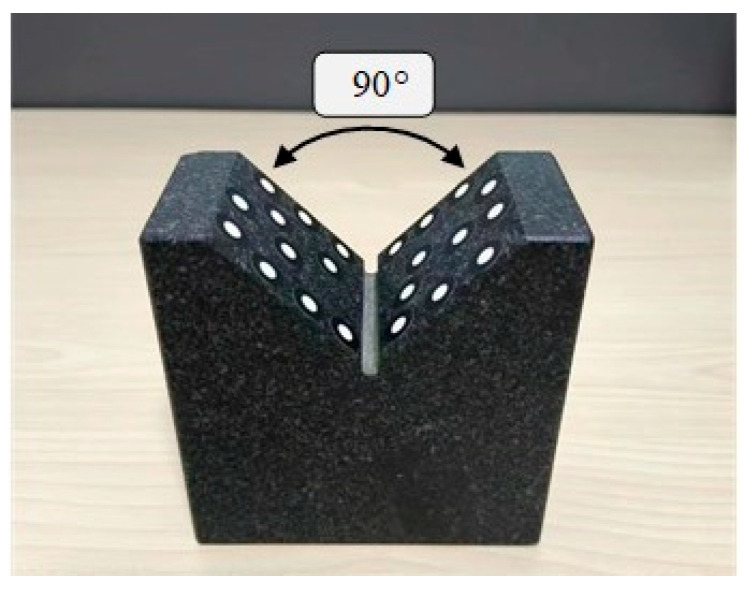
Marble V-block.

In the laboratory environment, the binocular stereo vision system is used to repeatedly measure the included angle of the V-block as shown in Figure 12 to verify the measurement accuracy of the attitude angle. The included angle of the marble V-block is 90° ± 0.01°. Circular non-coded marker points are pasted on both sides of the V-shaped surface. Then, the binocular stereo vision is used to measure the three-dimensional coordinates of these marker points, and these points are used to fit two planes that are close to vertical. Subsequently, the included angle between the two fitted planes is calculated, and the difference between this angle and the reference angle of 90° is calculated to obtain the measurement error of the attitude angle.

The angular error mainly comes from two aspects: (1) The reconstruction accuracy of the binocular stereo vision; (2) The change in the included angle caused by the pasting of the marker points. The measurement results are shown in the following table. The RMSE value of the measured angle is 0.065°, which indicates that the measurement accuracy of the attitude angle is better than 0.1°, see Table 4.

### 3.4. Comparison of Dynamic Pose Measurement Accuracy Using a Laser Total Station

**Figure 13:**
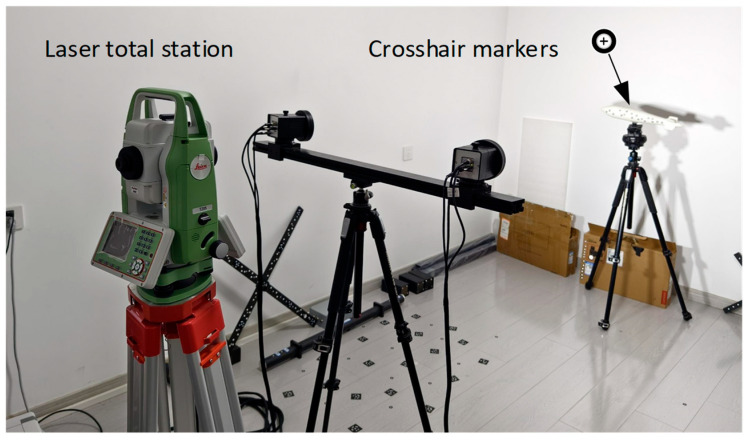
Setup of dynamic pose measurement using a laser total station.

To further evaluate the dynamic pose measurement accuracy, a laser total station (Leica TZ05) is employed as a reference tool. As shown in Figure 13, several crosshair markers, which are detectable by both the laser total station and the binocular stereo vision system, are strategically placed on the surface of the object. These markers serve as key reference points to ensure accurate and consistent measurements across both systems.

**Figure 14:**
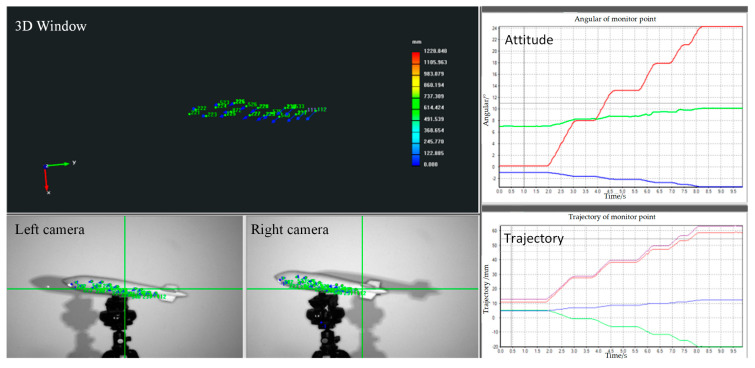
The dynamic pose measured by the binocular stereo vision system. In the attitude plot, the red, green, and blue lines correspond to roll, pitch, and yaw, respectively. In the trajectory plot, the red, green, blue, and purple lines represent the deformations along the x-axis, y-axis, z-axis, and their combination, respectively.

During the object’s motion, the binocular stereo vision system continuously measures the pose in real time, as depicted in Figure 14. The dynamic measurement process allows for the tracking of the object’s movement with high precision, enabling real-time updates of its position and orientation. When the object is stationary, both the laser total station and the binocular stereo vision system measure the pose independently. The results from both systems are then compared to assess the accuracy and reliability of the binocular stereo vision system.

Table 5 presents the attitude angle of a monitoring point on the object, as measured by both systems. It can be observed that the RMSE of the angle error is less than 0.1°. Table 6 displays the trajectory, where the RMSE is shown to be under 0.3 mm. This comparison offers valuable insights into the performance of the stereo vision system across various real-world scenarios, confirming its reliability and robustness for applications requiring precise dynamic pose measurement.

### 3.5. Experimental Measurement of the Dynamic Pose of the Landing Gear

**Figure 15:**
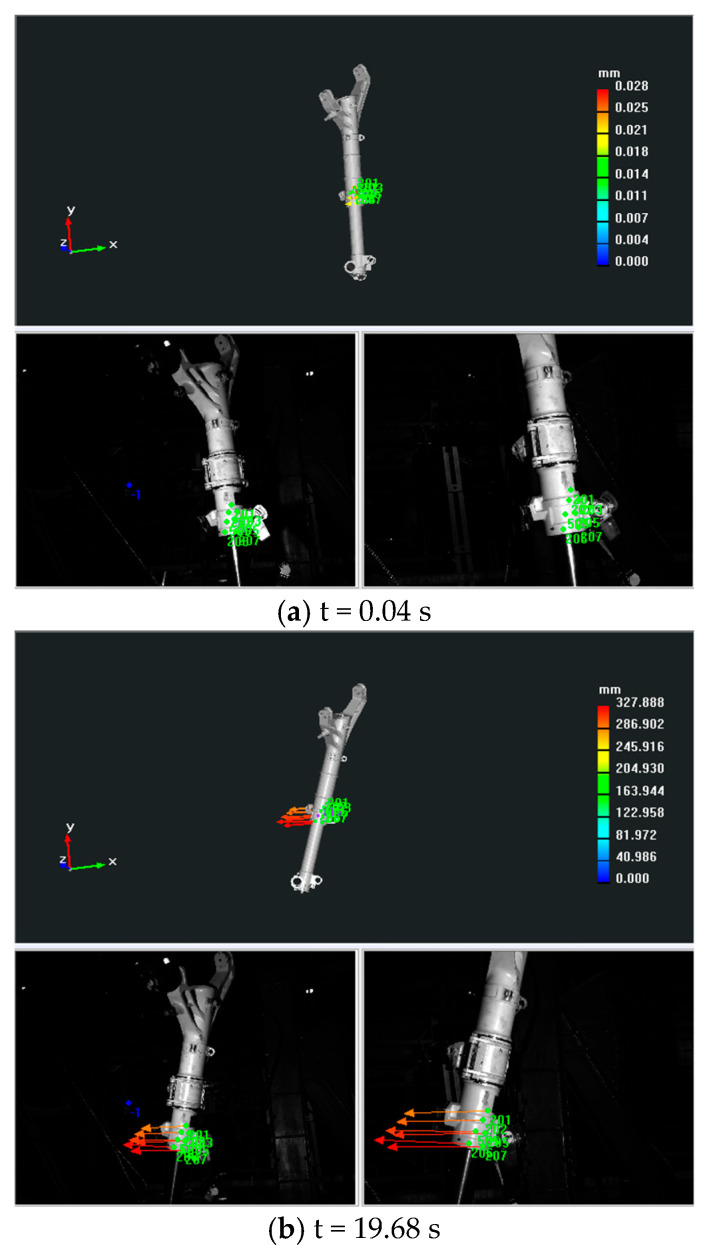

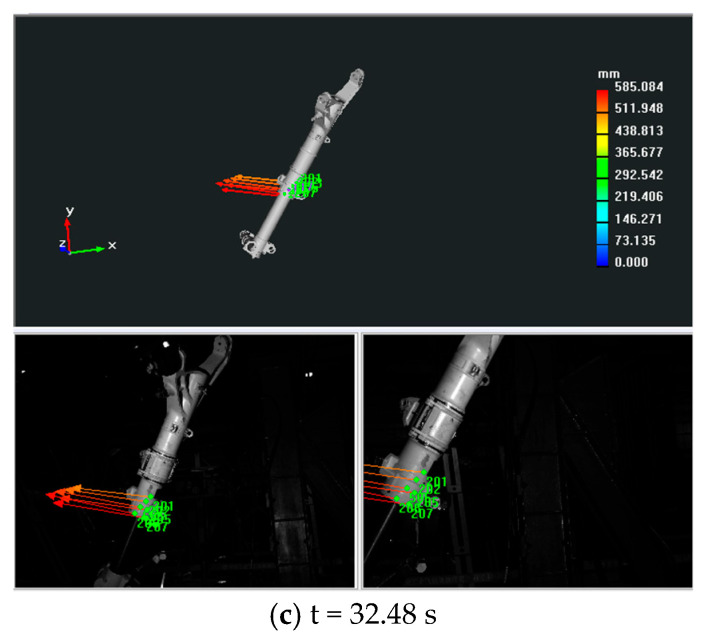
Imaginary and real posture diagram of a certain type of landing gear at different times.

**Figure 16:**
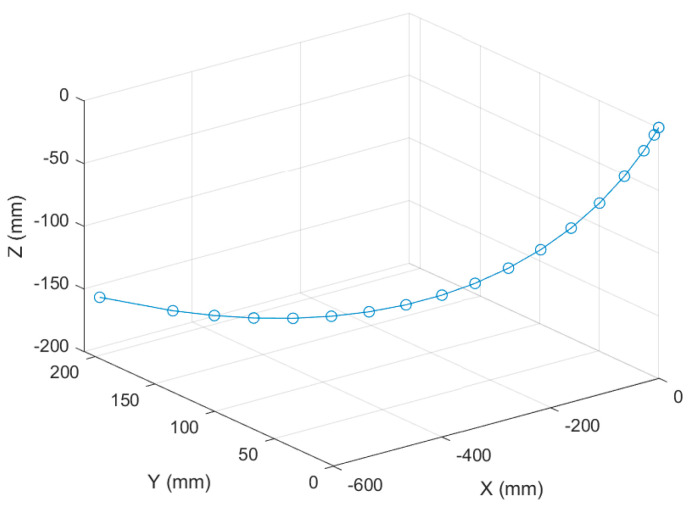
Motion Trajectory of a Certain Model of Landing Gear.

**Figure 17:**
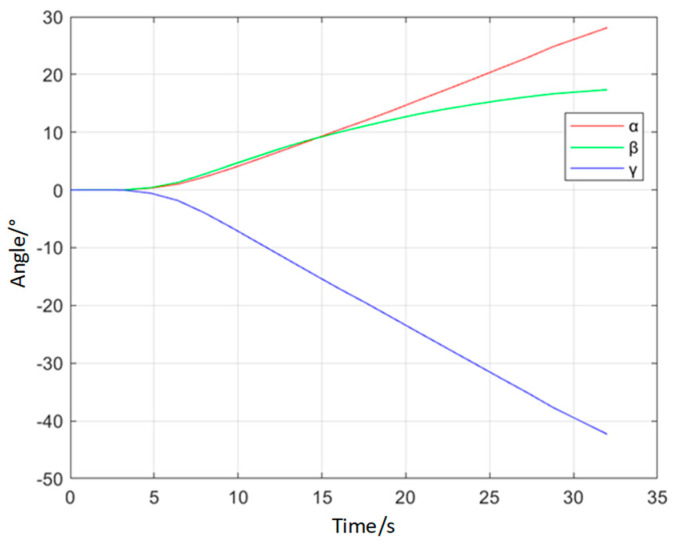
Pose Curve of a Certain Model of Landing Gear.

A dynamic pose measurement experiment was carried out on a certain type of landing gear. The acquisition and reconstruction frame rate of the binocular stereo vision is 50 Hz, and the size of the measurement field of view is 1.6 m × 1.1 m. Figure 15 shows the three-dimensional poses during the synchronous movement of the virtual and real models of the landing gear at different moments. Figure 16 shows the movement trajectory of a certain monitoring point on the landing gear, and Figure 17 shows the change in the attitude angle of this point over time. Table 7 lists the position and attitude angle values of this monitoring point at different moments.

During the structural strength test of the landing gear, the environmental conditions are generally complex. For example, factors like occlusion may be present, which adds to the uncertainty of the measurement process. The dynamic pose measurement method that combines virtual and real elements proposed in this paper only needs to observe a local area. By leveraging a small number of key points, it enables the synchronous movement of the CAD model and the physical model. In this way, the overall motion state and pose information can be obtained.

The results demonstrate that the proposed framework, which integrates close-range photogrammetry with binocular stereo vision, effectively addresses the critical challenge of occlusion in dynamic pose measurement. Unlike conventional methods that require dense optical markers and are susceptible to tracking failure, our approach establishes a stable correspondence between the physical landing gear and its CAD model by tracking a sparse set of key points. This strategy allows the CAD model to be driven synchronously with the physical motion, thereby enabling the reconstruction of the complete motion state even when partial occlusions occur. The achieved accuracy in real-time pose estimation under different working conditions confirms the robustness of this model-based approach.

Unlike the model-free, direct optical tracking methods of [5,7] that are susceptible to marker occlusion and data noise, our approach maintains stable pose estimation under these conditions. This capability for consistent performance in non-ideal scenarios makes it a more reliable and robust solution for the demanding environments common in landing gear research and development.

## 4. Conclusions

This paper proposes a visual measurement method for the dynamic pose of the landing gear that combines virtual and real elements. This method first establishes a measurement coordinate system on the virtual CAD digital model, which reduces the difficulty of establishing a measurement reference coordinate system on site. By combining close-range photogrammetry and binocular stereo vision technology, the coordinate system of the physical model of the landing gear is unified with the coordinate system of the virtual CAD digital model (i.e., the measurement coordinate system). Only by tracking a small number of key points can the virtual CAD model and the physical model be driven to move synchronously, so as to obtain the complete motion state of the landing gear during the test process.

Compared with existing methods, this method has the following advantages: (1) It has stronger environmental adaptability, effectively reducing the impact of adverse factors such as occlusion during the test process; (2) The strategy of combining virtual and real elements reduces the requirements for measurement equipment, improving the cost-effectiveness and efficiency of measurement; (3) Through abundant measurement data, the pose information of any point on the landing gear, including the center of mass, can be obtained. These advantages endow this method with higher reliability and flexibility in practical applications.

The current method relies on a sparse set of 6–10 key points. A primary limitation is its potential sensitivity to partial occlusions of these markers, which could interrupt tracking and pose estimation. To address this, a key direction for future research is the development of an optimal marker placement strategy that maximizes spatial distribution and ensures visibility throughout the expected motion path. Furthermore, we plan to investigate algorithmic improvements, such as incorporating redundant key points and developing robust pose estimation algorithms that can recover a stable solution even when a subset of markers is temporarily lost.

## Figures and Tables

**Table 1:** Calibration results of the internal and external parameters of the binocular cameras.

Parameter	Left Camera	Right Camera
*f*	4678.121	4669.225
$\left(x_{0},y_{0}\right)$	(45.704, −32.204)	(9.017, 18.523)
*K*_1_	−5.669975 × 10^−9^	−5.638504 × 10^−9^
*K*_2_	3.294475 × 10^−16^	3.146400 × 10^−16^
*K*_3_	−4.095361 × 10^−24^	−4.710919 × 10^−24^
*B*_1_	1.894295 × 10^−7^	1.147664 × 10^−7^
*B*_2_	3.925601 × 10^−8^	−1.101179 × 10^−7^
*E*_1_	−3.228971 × 10^−5^	9.913997 × 10^−5^
*E*_2_	1.220796 × 10^−5^	0.000162
***R***	$[\begin{array}{ccc} 0.891 & 0.081 & 0.447\\ -0.089 & 0.996 & -0.003\\ -0.446 & -0.037 & 0.894 \end{array}]$	
***T***	${ [\begin{array}{ccc} 843.637 & -33.150 & -160.207 \end{array}]}^{T}$	
Reprojection error	0.018	

**Table 2:** Reconstruction Results of the Scale in Close-Range Photogrammetry.

No.	Benchmark Value (mm)	Measurement Value (mm)	Absolute Error (mm)
1	832.436	832.459	−0.023
2	832.436	832.468	−0.032
3	832. 436	832.432	0.004
4	832. 436	832.425	0.011
5	832. 436	832.447	−0.011
6	832.561	832.543	0.018
7	832.561	832.542	0.019
8	832.561	832.558	0.003
9	832.561	832.564	−0.003
10	832.561	832.569	−0.008
RMSE			0.023

**Table 3:** Measured distances of marker points on the calibrated bar using the binocular stereo vision system.

Distance of Marker Points	Benchmark Value (mm)	Measurement Value (mm)	Absolute Error (mm)
1–2	70.431	70.369	0.062
1–3	130.526	130.468	0.058
1–4	198.487	198.520	−0.033
1–5	256.365	256.425	−0.06
1–6	321.349	321.404	−0.055
1–7	387.634	387.645	−0.011
1–8	577.682	577.652	0.03
1–10	702.516	702.588	−0.072
1–11	779.857	779.793	0.064
1–13	909.300	909.267	0.033
RMSE			0.073

**Table 4:** Angle Measurement Results of the V-Block.

No.	Benchmark Angle (°)	Measurement Angle (°)	Absolute Error (°)
1	90.000	89.941	0.059
2	90.000	89.934	0.066
3	90.000	89.938	0.062
4	90.000	89.931	0.069
5	90.000	89.933	0.067
RMSE			0.065

**Table 5:** Comparison of the Attitude Angle of a Monitoring Point on the Object.

No.	The Novel Method (°)			Laser Total Station (°)			Error (°)		
	α	β	γ	α	β	γ	α	β	γ
1	24.19	10.03	−3.5	24.28	10.07	−3.51	−0.09	−0.04	0.01
2	23.83	5.42	1.01	23.71	5.44	1.08	0.12	−0.02	−0.07
3	−15.58	−3.04	−1.45	−15.69	−3.11	−1.49	0.11	0.07	0.04
4	−32.71	−4.22	−3.23	−32.58	−4.18	−3.26	−0.13	−0.04	0.03
5	−2.35	31.65	−4.6	−2.3	31.72	−4.63	−0.05	−0.07	0.03
6	−2.31	24.39	−3.6	−2.27	24.48	−3.63	−0.04	−0.09	0.03
7	−1.66	12.62	−2.4	−1.56	12.52	−2.43	−0.1	0.1	0.03
8	3.45	−21.38	4.3	3.48	−21.22	4.35	−0.03	−0.16	−0.05
RMSE							0.09	0.08	0.04

**Table 6:** Comparison of the Trajectory of a Monitoring Point on the Object.

No.	The Novel Method (mm)			Laser Total Station (mm)			Error (mm)		
	X-Axis	Y-Axis	Z-Axis	X-Axis	Y-Axis	Z-Axis	X-Axis	Y-Axis	Z-Axis
1	14.66	−3.73	13.95	14.6	−3.7	13.9	0.06	−0.03	0.05
2	−19.50	6.00	−2.10	−19.7	5.8	−2.5	0.20	0.20	0.40
3	11.39	−30.60	9.50	11.3	−30.9	10.0	0.09	0.30	−0.50
4	14.62	−3.79	0.90	14.6	−3.7	0.7	0.02	−0.09	0.20
5	2.92	−64.20	−32.50	2.3	−64.1	−32.1	0.62	−0.1	−0.40
6	6.73	−47.80	−22.10	7.0	−47.4	−22.3	−0.27	−0.4	0.20
7	1.33	−22.70	2.80	1.1	−22.4	3.0	0.23	−0.3	−0.20
8	−1.31	51.60	−2.60	−1.0	51.5	−2.8	−0.31	0.10	0.20
RMSE							0.29	0.23	0.30

**Table 7:** Pose of a Monitoring Point on the Landing Gear.

Time/Second	Attitude Angle (Degree)			Trajectory (mm)		
	α	β	γ	X-Axis	Y-Axis	Z-Axis
0.00	0.00	0.00	0.00	0.00	0.00	0.00
1.68	0.00	0.04	0.00	0.00	0.00	0.01
3.28	0.02	0.02	−0.01	−0.15	0.00	−0.08
4.88	0.32	0.38	−0.56	−8.63	−0.159	−4.81
6.48	1.01	1.28	−0.82	−27.87	−0.124	−15.24
8.08	2.25	2.74	−3.97	−60.81	1.152	−32.24
9.68	3.72	4.33	−6.49	−99.17	4.54	−50.67
11.28	5.33	5.88	−9.15	−139.16	10.176	−68.4
12.88	6.99	7.41	−11.79	−178.53	17.771	−84.35
14.48	8.67	8.77	−14.44	−217.26	27.202	−98.65
16.08	10.39	9.99	−17.06	−254.77	38.178	−111.2
17.68	12.03	11.11	−19.55	−289.74	50.055	−121.7
19.28	13.76	12.17	−22.14	−325.56	63.895	−131.3
20.88	15.59	13.18	−24.75	−360.44	79.02	−139.4
22.48	17.37	14.03	−27.34	−394.19	95.271	−146.1
24.08	19.18	14.79	−29.92	−426.92	112.608	−151.5
25.68	21.03	15.51	−32.51	−458.78	131.02	−155.5
27.28	22.85	16.11	−35.05	−489.31	150.19	−158.4
28.88	24.84	16.67	−37.72	−519.99	171.05	−160.1
32.08	28.11	17.37	−42.30	−570.71	209.35	−160.1

## Data Availability

The data presented in this study are available from the corresponding authors upon reasonable request.
